# Andexanet alfa for the reversal of the low-molecular-weight heparin enoxaparin

**DOI:** 10.1016/j.rpth.2025.103191

**Published:** 2025-09-23

**Authors:** Thijs F. van Haaps, Alexander P. Benz, Lizhen Xu, Saskia Middeldorp, John W. Eikelboom, Truman J. Milling, Mark Crowther, Lisa Holer, Stephan Nolan, Per Ladenvall, Genmin Lu, Michiel Coppens

**Affiliations:** 1 Department of Vascular Medicine, Amsterdam University Medical Centers, location University of Amsterdam, Amsterdam, the Netherlands; 2 Amsterdam Cardiovascular Sciences, Pulmonary Hypertension & Thrombosis, Amsterdam, the Netherlands; 3 Department of Cardiology, University Medical Center Mainz, Johannes Gutenberg-University, Mainz, Germany; 4 Population Health Research Institute, McMaster University, Hamilton, Ontario, Canada; 5 Department of Internal Medicine, Radboud University Medical Center, Nijmegen, the Netherlands; 6 Department of Medicine, McMaster University, Hamilton, Ontario, Canada; 7 Seton Dell Medical School Stroke Institute, Dell Medical School, University of Texas at Austin, Austin, Texas, USA; 8 AstraZeneca, Gothenburg, Sweden; 9 Alexion, AstraZeneca, Dublin, Ireland; 10 Alexion, AstraZeneca, South San Francisco, California, USA

**Keywords:** andexanetalfa, enoxaparin, heparin, low-molecular-weight, hemorrhage, intracranial hemorrhages, venous thromboembolism

## Abstract

**Background:**

Andexanet alfa (andexanet) is the only approved reversal agent for patients with acute major bleeding during apixaban or rivaroxaban treatment. Its mechanism suggests it may also reverse the effects of low-molecular-weight heparin.

**Aim:**

To evaluate the effects of andexanet in healthy volunteers and in patients with acute major bleeding on enoxaparin.

**Methods:**

In the first study, healthy volunteers received enoxaparin 1 mg/kg twice daily for ≥3 doses and were randomized to receive either andexanet or placebo in high- or low-dose regimens, given 3 hours (high dose) or 8 hours (low dose) after the last dose. In the second study (Andexanet Alfa, a Novel Antidote to the Anticoagulant Effects of FXa Inhibitors 4), dosing depended on timing and amount of last enoxaparin dose: high dose if > 40 mg or <8 hours since last dose (or unknown), low dose if ≤ 40 mg or ≥8 hours. The high dose consisted of an 800 mg bolus followed by 960 mg >2 hours, while low dose consisted of a 400 mg bolus followed by 480 mg >2 hours. The primary outcome was change in anti-Xa activity. Hemostatic efficacy was assessed in patients with confirmed bleeding and baseline anti-Xa ≥ 0.25 IU/mL.

**Results:**

In the first study, 24 volunteers received andexanet (12 at a high dose and 12 at a low dose), and 7 received a placebo. Median anti-Xa activity decreased by 83% (0.92-0.17 IU/mL) in the high-dose cohort, and by 73% (0.78-0.22 IU/mL) in the low-dose cohort. In the second study, 22 patients received andexanet; anti-Xa activity decreased by 75% (95% CI, 67%-79%; 0.48-0.14 IU/mL). Good/excellent hemostasis was achieved in 14 of 16 assessable patients (88%).

**Conclusion:**

In enoxaparin-treated subjects, andexanet reduced anti-Xa activity and achieved effective hemostasis in 88% of patients. Results align with findings of patients using apixaban, rivaroxaban, or edoxaban.

## Essentials

• Andexanet alfa neutralizes direct FXa inhibitors and binds heparin-antithrombin.
• We studied andexanet alfa in healthy subjects and in bleeding patients treated with enoxaparin.
• Andexanet alfa swiftly lowered anti-Xa activity and led to good bleeding control.
• Our findings are consistent with results of patients using apixaban, rivaroxaban, or edoxaban.

## 1 Introduction

Optimal management of acute major bleeding in patients treated with direct oral anticoagulants is necessary to improve clinical outcomes. Andexanet alfa (andexanet), a modified, recombinant, inactive form of human factor (F)Xa, was developed as a reversal agent to neutralize the anticoagulant effects of direct and indirect FXa inhibitors. Its effects have been evaluated in the Andexanet Alfa, a Novel Antidote to the Anticoagulant Effects of FXa Inhibitors (ANNEXA) studies [1,2]. Andexanet received conditional approval from the United States Food and Drug Administration and the European Medicines Agency as the first specific reversal agent for FXa inhibitors, apixaban and rivaroxaban, and additionally received full approval in Japan for reversal of all FXa inhibitors, including edoxaban [3]. After these conditional approvals, the ANNEXA-4 study continued, including a total of 479 patients with acute major bleeding within 18 hours after administration of a FXa inhibitor [4]. The administration of andexanet was associated with a swift reduction in anti-Xa activity. Good or excellent hemostasis was achieved in 80% of patients, 16% of patients died, and 10% had a thrombotic event.

In addition to the ability of andexanet to bind and sequester direct FXa inhibitors, andexanet also binds heparin-bound antithrombin, providing a rationale for the reversal of not only direct but also indirect FXa inhibitors (eg, unfractionated [UFH] and low-molecular-weight heparin [LMWH] and fondaparinux). Although andexanet is not licensed for the reversal of enoxaparin, preclinical, healthy volunteer, and clinical data support the effective reversal of heparins (UFH and LMWH) and fondaparinux, which indirectly inhibit FXa through antithrombin.

Here, we present data on the effect of andexanet on anti-Xa activity in healthy volunteers treated with enoxaparin, as well as the outcomes of a subgroup of patients with acute major bleeding treated with enoxaparin and enrolled in the ANNEXA-4 study.

## 2 Methods

The efficacy and safety of andexanet for the reversal of the LMWH enoxaparin were evaluated in 2 studies. First, the effects of andexanet or placebo on laboratory parameters were evaluated in healthy volunteers treated with enoxaparin. Second, the effects of andexanet on laboratory and clinical outcomes were evaluated in a subgroup of patients treated with enoxaparin who presented with acute major bleeding and enrolled in the ANNEXA-4 study [4].

### 2.1 Healthy volunteer study

#### 2.1.1 Study design

The healthy volunteer study was a randomized, double-blind study involving subjects dosed at a steady state with enoxaparin, designed to evaluate the degree to which andexanet reverses enoxaparin-induced anticoagulation (ClinicalTrials.gov, NCT03083704). The study protocol was approved by the institutional review board at study location, and all participants provided written informed consent before study enrollment. All subjects underwent a full physical examination, electrocardiography, and laboratory tests for the purpose of screening and assessment of eligibility (eg, testing for FV Leiden, hepatitis B/C, and HIV screening). Key inclusion criteria were an age between 18 and 75 years, body mass index between 19 and 30 kg/m^2^, a body weight of ≥ 50 kg, and a blood pressure of < 160/90 mm Hg. Subjects with well-controlled, chronic, and stable conditions (eg, hypertension, non–insulin-dependent diabetes mellitus, or hypothyroidism) were eligible for inclusion based on the clinical judgment of the investigator. Key exclusion criteria were previous use of andexanet or participation in the current study, history of abnormal bleeding or signs or symptoms of active bleeding, or past or current medical history of thrombosis. Details regarding the inclusion and exclusion criteria, as well as the included laboratory tests before enrollment, can be found in Supplementary Texts S1 and S2. After screening and enrollment, subjects were domiciled at the study site for 8 days and received enoxaparin at a dose of 1 mg/kg twice daily to reach steady state. On day 4, randomization took place, after which subjects were assigned to receive andexanet high dose, andexanet low dose, or placebo. Andexanet or placebo was administered as a bolus injection followed by a continuous infusion for 120 minutes. The high dose consisted of a bolus injection of 800 mg, followed by a 120-minute infusion of 960 mg (8 mg/min). The low dose consisted of a 400 mg bolus, followed by a 120-minute infusion of 480 mg (4 mg/min). The high-dose group received andexanet 3 hours after the last dose of enoxaparin, while the low-dose group received andexanet 8 hours after the last dose of enoxaparin. A lyophilized placebo product (100 mg/vial) with the same appearance and container closure, containing all ingredients of the active product except andexanet, served as the placebo control. The same timing of administration was used as described for the andexanet group. Study subjects were discharged from the clinical site on day 8 and followed for safety through day 32 via telephone.

#### 2.1.2 Clinical and laboratory outcomes

The primary outcome of the healthy volunteer study was a relative change in anti-Xa activity from baseline (before administration of andexanet or placebo) to nadir (the smallest value from 10 minutes prior to end of andexanet or placebo bolus until 5 minutes after end of infusion). The anti-Xa activity was measured using a commercial kit with validated modifications, including an additional dilution step, to prevent dissociation of the andexanet-Xa inhibitor complex (Coamatic Heparin, DiaPharma) [5]. Anti-Xa activity was measured using the STA Compact analyzer from Stago. Calibration was performed with enoxaparin standards prepared in pooled normal plasma. A 100 IU/mL working solution of enoxaparin was prepared from a 10,000 IU/mL stock solution and further diluted to a final solution of 2.0 IU/mL. Assay standards and controls were prepared from this solution as detailed in Supplementary Tables S2 and S3. The secondary outcome was a change in thrombin generation as measured by endogenous thrombin potential (ETP). Adverse events (AEs) were collected and included development of antibodies to andexanet, FX, and FXa. Antibodies were measured at baseline and day 30 using validated immunoassays with electrochemiluminescent detection at a bioanalytical laboratory [2,4]. For any sample confirmed positive for antibodies against andexanet, the potential for neutralizing antibody activity was further assessed by measuring the functional activity of andexanet in plasma [1,4,5]. Thrombin generation was measured using a calibrated automated thrombogram according to the manufacturer’s instructions (Diagnostica Stago). The assay was performed using a commercial platelet-poor plasma Reagent (Diagnostica Stago) containing 5 pM recombinant tissue factor and 4 μM phospholipids for initiation. According to the manufacturer’s recommendation, no modifications were used. ETP was prospectively chosen as the endpoint for thrombin generation.

#### 2.1.3 Statistical analysis and sample size

Baseline data were summarized by cohort using descriptive statistics. A 2-sample Wilcoxon rank-sum test was used to compare the anti-Xa activity between andexanet and placebo in the healthy volunteers. Nine-five percent CIswere calculated using the Hodges–Lehman estimate with a 2-sided significance level of 5%. For the safety outcomes, the number of events, number of subjects, and the percentage of subjects who experienced at least 1 AE were presented.

Sample size was based on the following power calculation. For within-cohort analyses, a total of 9 subjects (6 andexanet and 3 placebo) would yield at least 90% power to detect a difference in anti-Xa activity between andexanet and placebo at a significance level of .05 (2-sided) using the Wilcoxon rank-sum test. All of the underlying assumptions were supported by data from other studies [1,6].

### 2.2 Patients with acute major bleeding

#### 2.2.1 Study design

The ANNEXA-4 study was a multicenter, prospective, open-label, single-group study that included patients treated with a FXa inhibitor for acute major bleeding [2,4]. The study was approved by institutional review boards at each center; all patients provided written informed consent. The ANNEXA-4 study is registered at ClinicalTrials.gov (NCT02329327). Details regarding the inclusion and exclusion criteria can be found in Supplementary Text S3. In summary, patients were included if they were at least 18 years old, presented with acute major bleeding, and had received an oral FXa inhibitor or enoxaparin within 18 hours. For the present analysis, all patients treated with enoxaparin at a dose of ≥ 1 mg/kg/d were included. The results of 22 patients receiving enoxaparin have been included in the published full study report [4]. Patients were excluded if they were scheduled for surgery within 12 hours after presentation (except for minimally invasive surgery or procedures, including burr holes for intracranial hemorrhage [ICH]). In patients with ICH, those with a Glasgow Coma Scale score <7 or an estimated intracerebral hematoma volume of > 60 mL were also excluded.

The dose of andexanet was based on the time and dose of the last enoxaparin administration. A low dose was given if the last administered dose was ≤ 40 mg or if the last dose was given ≥8 hours before presentation. A high-dose regimen was given if the time since last dose of enoxaparin was <8 hours or unknown, and if the last administered dose was > 40 mg or unknown. The same high- and low-dose regimens were used as in the healthy volunteer study described above.

Enrolled patients were assessed at multiple time points: within 3 hours prior to the administration of andexanet (baseline); at the end of bolus administration (15-30 minutes after initiation); at the end of the 2-hour infusion; at 4 hours, 8 hours, and 12 hours after the end of the infusion; and at 3 days and 30 days. Multiple blood samples, including anti-Xa activity, were obtained during a 12-hour period at the same time points as the clinical assessments. For patients with ICH, computed tomography or magnetic resonance imaging of the head was performed within 3 hours before the initiation of the andexanet bolus and at 1 hour, 12 hours, and 30 days after the end of the infusion.

#### 2.2.2 Clinical and laboratory outcomes

The ANNEXA-4 study had 2 coprimary efficacy outcomes: a relative change from baseline in anti-Xa activity after andexanet treatment, and the percentage of patients achieving good or excellent hemostatic efficacy 12 hours after the andexanet infusion. Hemostatic efficacy was assessed using a 3-point system (excellent, good, or poor/none) by an adjudication committee on the basis of prespecified criteria for ICH, visible bleeding, and the different manifestations of nonvisible bleeding (Supplementary Table S1). Secondary outcomes included a change in thrombin generation, as measured by ETP, from baseline to postandexanet peak. Safety outcomes included thrombotic events, all-cause mortality up to 30 days, and the development of antibodies to native FX, FXa, and andexanet. Antibody measurements were the same as those in the healthy volunteer study described above. As implemented in Protocol Amendment 4, a modified Bethesda assay was used to assess the potential presence of neutralizing antibodies to endogenous FX/FXa.

#### 2.2.3 Statistical analysis

Baseline data of all participants were summarized using descriptive statistics. The coprimary efficacy outcomes were analyzed by assessing the percent change from baseline in anti-Xa activity, with a 2-sided nonparametric CI for the median. Next, the proportions of patients with effective hemostasis were evaluated using the 3-point system described above and are presented with a binomial 95% CI. For the analyses of efficacy and safety, 2 cohorts were created. The safety population included all patients who received andexanet, while the efficacy population included only enoxaparin-treated patients in whom the baseline anti-Xa activity was later determined to be ≥ 0.25 IU/mL and the acute major bleeding episode was adjudicated to meet the study criteria. Safety outcomes were reported as the number of events and the proportion of subjects who experienced at least 1 event.

## 3 Results

### 3.1 Healthy volunteer study

A total of 31 subjects were included, with 16 subjects allocated to the high-dose regimen (12 to andexanet and 4 to placebo) and 15 to the low-dose regimen (12 to andexanet and 3 to placebo). The treatment groups were balanced with respect to baseline characteristics (Table 1). There were no deaths or other AEs in the study. No antibodies to andexanet, FX, or FXa were detected.

**Table 1:** Baseline characteristics of subjects in the healthy volunteer study.

Characteristic	High-dose andexanet (*n* = 16)		Low-dose andexanet (*n* = 15)	
	Andexanet (*n* = 12)	Placebo (*n* = 4)	Andexanet (*n* = 12)	Placebo (*n* = 3)
Age (y)				
Mean (SD)	40.6 (10.3)	41.5 (7.2)	41.6 (15.7)	38.3 (14.2)
Minimum, maximum	23, 57	35, 50	26, 67	22, 48
Women, *n* (%)	5 (42)	2 (50)	7 (58)	2 (67)
Race, *n* (%)				
Asian	3 (25)	3 (75)	7 (58)	2 (67)
Black or African American	3 (25)	1 (25)	5 (42)	1 (33)
White	6 (50)	0	0	0
Weight (kg), mean (SD)	72.2 (13.77)	74.2 (6.35)	77.9 (12.84)	69.8 (12.02)
Body mass index (kg/m^2^), mean (SD)	24.9 (3.21)	26.5 (2.44)	26.7 (2.58)	27.2 (1.82)

**Figure 1:**
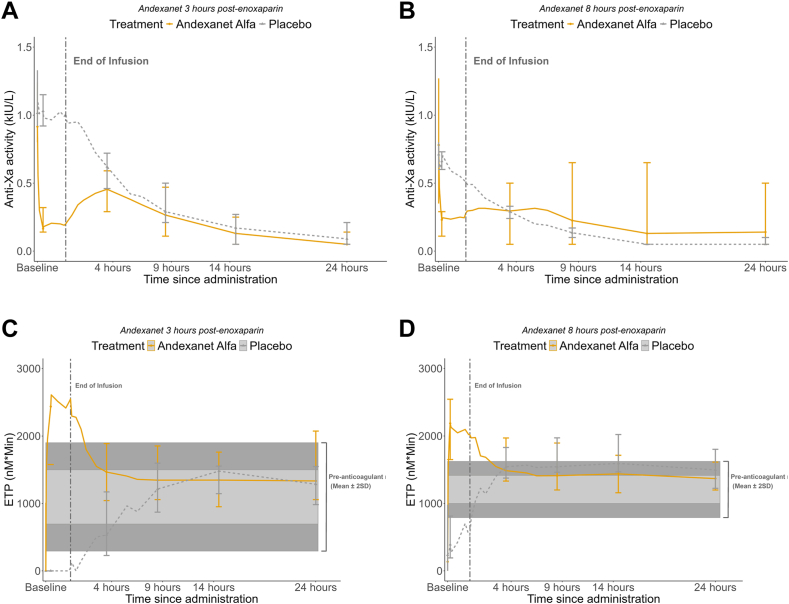
Effects of andexanet or placebo on healthy volunteers. All values are presented as medians and ranges (A and B), anti-Xa activity, and (C and D) endogenous thrombin potential (ETP) over time. The vertical dashed line in each panel indicates the end of infusion. In C and D, the shaded areas indicate the preanticoagulant ETP ± 1 SD (light gray) or ±2 SDs (dark gray) measured prior to enoxaparin dosing. The lower limit of quantitation for anti-Xa activity was 0.1 IU/mL. ANNEXA-4, Andexanet Alfa, a Novel Antidote to the Anticoagulant Effects of FXa Inhibitors-4; kIU, kilo international unit.

In the subjects randomized to the high-dose cohort, the median anti-Xa activity decreased by 83% from 0.92 IU/mL at baseline to 0.17 IU/mL at nadir. The corresponding values for placebo were 1.02 IU/mL at baseline and 0.93 IU/mL at nadir (14% decrease; Figure 1A). In the low-dose cohort, the median anti-Xa activity decreased by 73% from 0.78 IU/mL at baseline to 0.22 IU/mL at the end of the bolus administration. The corresponding values for placebo were 0.71 IU/mL at baseline and 0.49 IU/mL at nadir (26% decrease; Figure 1B). Both high and low doses of andexanet significantly reduced enoxaparin anti-Xa activity compared with placebo (*P* = .01 and *P* = .02, respectively).

In the subjects in the high-dose cohort randomized to andexanet, median ETP increased by 2641 nM ∗ min (range, 1387-3476) at the end of the bolus administration. In subjects randomized to placebo, ETP increased by 121 nM ∗ min (range, 0-227). In the low-dose cohort, median ETP increased by 1966 (range, 1273-2512) in subjects randomized to andexanet and by 538 nM ∗ min (range, 362-674) in subjects randomized to placebo. Both high and low doses of andexanet significantly increased ETP compared with placebo (*P =* .01 and *P =* .02, respectively).

### 3.2 Patients with acute major bleeding

**Figure 2:**
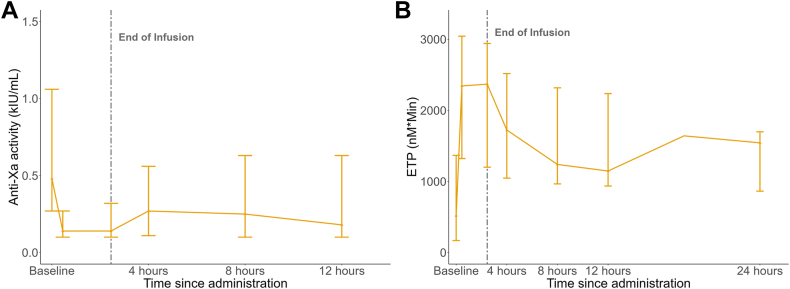
Effect of andexanet alfa on patients with acute major bleeding treated with enoxaparin in the ANNEXA-4 study. All values are presented as medians and ranges (A), anti-Xa activity, and (B) endogenous thrombin generation (ETP). In both A and B, the vertical dashed line indicates the end of infusion. ANNEXA-4, Andexanet Alfa, a Novel Antidote to the Anticoagulant Effects of FXa Inhibitors-4; kIU, kilo international unit.

The ANNEXA-4 study included 22 patients treated with enoxaparin (safety population). Mean age was 68 years, and 10 (46%) of the participants were women (Table 2). The most common indications for anticoagulation were venous thromboembolism (64%) and atrial fibrillation (27%). The primary site of bleeding was intracranial (50%), followed by gastrointestinal (32%). Seventeen patients (77%) had an anti-Xa ≥ 0.25 IU/mL (efficacy population). In the efficacy population, the median anti-Xa activity decreased by 75% (95% CI, 67%-79%) from 0.48 IU/mL at baseline to 0.14 IU/mL at the end of the bolus administration (Table 3). At 4, 8, and 12 hours after andexanet continuous infusion, the median anti-Xa activity was reduced from baseline by 46%, 57%, and 63%, respectively (Figure 2A). Of the 17 patients in the efficacy analysis, 16 could be evaluated for hemostatic efficacy. One patient withdrew consent on day 1, and hemostatic efficacy could therefore not be assessed. Effective hemostasis was achieved in 14 patients (88%). Stratified by bleeding location, effective hemostasis was achieved in 8 of the 10 patients with ICH (80%) and in all 6 patients with extracranial bleeding (100%) (Table 4). Median ETP was available for 14 patients in the efficacy population and increased from 519 nM ∗ min at baseline to 2344 nM ∗ min at the end of bolus administration.

**Table 2:** Baseline characteristics of patients with acute major bleeding (ANNEXA-4).

Characteristic	ANNEXA-4 patients (*N* = 22)
	*n* (%)^a^
Age (y), mean (SD)	68.0 (9.4)
Minimum, maximum	53, 58
Women	10 (46)
Weight (kg), mean (SD)	80.1 (20.3)
Body mass index (kg/m^2^), mean (SD)	27.6 (6.4)
Estimated creatinine clearance^b^	
< 30 mL/min	0 (0)
30-59.9 mL/min	7 (32)
≥ 60 mL/min	15 (68)
Primary indication for anticoagulation	
Venous thromboembolism	14 (64)
Atrial fibrillation	6 (27)
Other	2 (9)
Medical history	
Deep vein thrombosis	9 (41)
Diabetes mellitus	9 (41)
Pulmonary embolism	8 (36)
Atrial fibrillation	8 (36)
Stroke	6 (27)
Myocardial infarction	2 (9)
Heart failure	2 (9)
Primary site of bleeding	
Intracranial	11 (50)
Gastrointestinal	7 (32)
Other	4 (18)
Enoxaparin	
Total daily dose (mg/kg bodyweight), mean (SD)	1.6 (0.5)
Once daily dose	4 (18)
Twice daily dose	18 (82)
Time from last dose to andexanet (h), mean (SD)	12.5 (4.8)
Time from ED presentation to andexanet bolus (h), mean (SD)	5.9 (2.1)

ANNEXA-4, Andexanet Alfa, a Novel Antidote to the Anticoagulant Effects of FXa Inhibitors-4; ED, emergency department.

a Unless otherwise indicated.

b Creatinine clearance was estimated according to the Cockcroft–Gault formula.

**Table 3:** Effects of Andexanet on anti-Xa activity, and Endogenous Thrombin Potential (ETP) in healthy subjects and ANNEXA-4 patients.

Variable	Healthy subjects		ANNEXA-4 patients
	High-dose andexanet	Low-dose andexanet	Efficacy population
Percent change in anti-Xa activity baseline to:			
End of bolus^a^	−82.5	−73.0	−73.9 (−79.2, −66.7)
First time point^a^	−51.2	−62.0	−48.9 (−56.8, −42.5)
Second time point^a^	−70.6	−70.4	−53.1 (−62.0, −38.2)
Third time point^a^	−85.3	−80.0	−60.4 (−73.0, −50.0)
Anti-Xa activity (kIU/mL)			
Baseline value	0.915 (0.80, 1.24)	0.780 (0.35, 1.27)	0.48 (0.27, 1.06)
End of bolus	0.165 (0.14, 0.32)	0.220 (0.11, 0.29)	0.14 (0.10, 0.32)
First time point	0.455 (0.29, 0.59)	0.295 (0.05, 0.50)	0.27 (0.11, 0.56)
Second time point	0.265 (0.11, 0.47)	0.225 (0.05, 0.65)	0.25 (0.10, 0.63)
Third time point	0.130 (0.05, 0.25)	0.130 (0.05, 0.65)	0.18 (0.10, 0.63)
ETP (nM ∗ min)			
Preenoxaparin administration	1022.33 (564.36, 1802.63)	1209.59 (847.91, 1672.49)	NA
Baseline value	0.000 (0.000, 1010.65)	1479.835 (0.00, 594.31)	518.87 (168.19, 3044.15)
End of bolus	2437.820 (1577.14, 3402.73)	2187.080 (1649.75, 2543.96)	2343.63 (1322.36, 3044.15)
First time point	1465.385 (1041.70, 1887.84)	1486.185 (1330.75, 1970.50)	1723.57 (1047.23, 2518.76)
Second time point	1345.280 (1056.98, 1852.38)	1410.655 (1199.05, 1895.39)	1240.18 (966.02, 2317.19)
Third time point	1346.015 (950.67, 1760.58)	1437.795 (1157.95, 1713.33)	1147.34 (936.21, 2235.80)

Effects of andexanet on anti-Xa activity and endogenous thrombin potential in healthy subjects and ANNEXA-4 patients.

Percent change in anti-Xa activity is presented as means with corresponding 95% CIs when available, and anti-Xa activity and ETP are presented as medians with minimum and maximum values.

ANNEXA-4, Andexanet Alfa, a Novel Antidote to the Anticoagulant Effects of FXa Inhibitors 4; ETP, endogenous thrombin potential; kIU, kilo international unit; NA, not available.

a The first, second, and third time points were performed after 3.5, 8.5, and 14.5 hours in the healthy volunteer study and after 4, 8, and 12 hours in bleeding patients for ANNEXA-4.

**Table 4:** Clinical outcomes of patients with acute major bleeding on enoxaparin treated with andexanet alfa.

Variable	Effective hemostasis	Thrombotic complication	Death
	*n*/*N* (%)	*n*/*N* (%)	*n*/*N* (%)
All bleeding	14/16 (88)	1/22 (5)	2/22 (9)
Site of bleeding			
Intracranial	8/10 (80)	0/11 (0)	2/11 (18)
Nonintracranial	6/6 (100)	1/11 (9)	0/11 (0)

Two patients (9%) in the safety population died within 30 days; both had suffered an ICH, and death was deemed to be unrelated to the study medication. One of these patients, an 80-year-old, died 19 days after receiving andexanet due to a noncardiovascular event. The other patient, a 59-year-old, died 23 days after receiving andexanet due to a cardiovascular event not related to bleeding. No venous thromboembolic events were reported after receiving andexanet. One patient (5%) developed a stroke of uncertain classification 7 days after andexanet administration and before restart of anticoagulation. In 14 patients (64%), anticoagulation was restarted. No antibodies to andexanet, FX, or FXa were detected.

## 4 Discussion

In healthy volunteers and patients with acute major bleeding receiving therapeutic-dose enoxaparin, andexanet significantly reduced the anti-Xa activity by 73% to 83%. This reduction led to a swift increase in thrombin generation, as assessed by ETP, to levels of unanticoagulated persons or higher. In patients with acute major bleeding treated with andexanet, 88% of patients had effective hemostasis, 5% developed a thrombotic complication, and 9% of patients died. The observed results in enoxaparin-treated patients with acute major bleeding are consistent with findings on the use of andexanet as reversal agent for the oral FXa inhibitors rivaroxaban, apixaban, and edoxaban [1,4,7].

A partial return of anti-Xa activity 2 hours after the end of infusion of andexanet suggests a temporary reversal effect of enoxaparin, similar to observations in patients who receive andexanet for oral FXa-associated acute major bleeding. In contrast, ETP levels increased to those of nonanticoagulated persons up until the last measurement, 24 hours after andexanet administration, suggesting an extended restoration of physiological hemostasis after the administration of andexanet.

Andexanet restored ETP to levels higher than baseline, on average. It remains uncertain whether this effect is associated with thrombotic complications that have occurred in andexanet-treated patients, as seen in the recently published ANNEXA-I trial, which randomized patients to either andexanet or standard of care (prothrombin complex concentrate in most [8]). The above baseline values persisted for only 4 hours, while in ANNEXA-4, 68% of thrombotic complications were observed >7 days after andexanet. Previously published biomarker analyses of the ANNEXA-4 study showed that andexanet administration is associated with reduced circulating tissue factor pathway inhibitor activity, which may also play a role in the observed thrombotic complications. The other main potential cause of thrombotic complications after bleeding and reversal is the temporary or definitive interruption of anticoagulation [9].

Although the effect of andexanet on other LMWHs or fondaparinux has not been tested, we expect that andexanet will be similarly effective in reversing the effects because it shares a similar mode of action with enoxaparin, ie, catalyzing the inhibition of coagulation by binding to antithrombin [10]. The use of andexanet as reversal agent for both LMWH and UFH has been suggested by *in vitro* studies, which concluded that andexanet nearly fully reverses UFH and neutralizes its anticoagulant activity [11].

An alternative to andexanet for reversal of UFH heparin and LMWH is protamine sulfate or chloride. In healthy volunteers, protamine completely reverses anti-II activity, but only partly reverses anti-Xa activity [12,13]. The extent of reversal of anti-Xa activity differs among the different types of LMWH, ranging from 54% to 86% [14]. However, there is very little clinical data on the outcome of patients with acute major bleeding managed with protamine, and its use can lead to severe hypotension and anaphylaxis-like reactions. Finally, protamine is ineffective in the reversal of the pentasaccharide fondaparinux, which can be reversed by andexanet.

A strength of our report is the strict prospective design and execution of the 2 studies for both the healthy volunteers and patients with acute major bleeding. Study outcomes were clearly defined and measured at predefined time points.

Some limitations apply. The ANNEXA-4 study included only a modest number of patients treated with enoxaparin, although this still yielded an estimate of effective hemostasis and anti-Xa reduction. ANNEXA-4 excluded ICH patients with a Glasgow Coma Scale score <7, an estimated ICH volume of > 60 mL, or <1 month of expected survival. However, it is unlikely that the prognosis of these patients can be favorably modified by the administration of a reversal agent. Finally, we studied effects of andexanet on only 1 type of LMWH preparation. Future studies could further explore the effect of andexanet on reversal and outcomes in patients treated with other LMWH preparations or fondaparinux [15].

In conclusion, this analysis of healthy volunteers and patients with acute major bleeding using therapeutic-dose enoxaparin shows that andexanet reverses its anticoagulant effect. The results suggest a similar clinical efficacy and safety profile of andexanet as previously observed in patients treated with oral FXa inhibitors, including rivaroxaban, apixaban, and edoxaban. The use of andexanet as reversal therapy for other LMWHs warrants further evaluation.
