# Supplementary material for: Andexanet alfa for the reversal of the low-molecular-weight heparin enoxaparin
Source: Res Pract Thromb Haemost. 2025 Sep 23;9(7):103191. doi: 10.1016/j.rpth.2025.103191 (PMC12552982; doi:10.1016/j.rpth.2025.103191)
Supplement: Supplementary Material [file mmc1.docx]

**Supplementary list and tables**

**Supplementary Text 1: Inclusion and exclusion criteria healthy volunteer study:**

To be eligible for study enrollment, potential study patients must satisfy all of the following

inclusion criteria:

1) Must have been in reasonably good health as determined by the PI based on medical history, full physical examination (including blood pressure and pulse rate measurement), 12-lead ECG, and clinical laboratory tests. Subjects with well-controlled, chronic, stable conditions (e.g., controlled hypertension, non–insulin-dependent diabetes, osteoarthritis, hypothyroidism) could have been enrolled based on the clinical judgment of the PI.

2) Must have been between the ages of 18 and 75 years, inclusive, at the time of signing the ICF.

3) Agreed to have any dietary or nutritional supplements reviewed by the PI and potentially held during the study if advised by the PI. Standard multivitamin and mineral supplementation were permitted.

4) Agreed to comply with the contraception and reproduction restrictions of the study.

○ Men whose sexual partner was of childbearing potential and/or who were not monogamous must have been using two acceptable methods of contraception, at least one of which must have been a barrier method (e.g., spermicidal gel plus condom), for the entire duration of the study and for at least 1 month following study drug administration; and men must have refrained from attempting to father a child or donating sperm in the 1 month following the study drug administration. Periodic abstinence (e.g., calendar, ovulation, symptom-thermal, post-ovulation methods) and withdrawal were not acceptable methods of contraception;

○ Men who reported surgical sterilization (e.g., bilateral vasectomy) must have had the procedure at least 6 months before study drug administration;

○ Surgical sterilization procedures were to be supported with clinical documentation and noted in the Relevant Medical History/Current Medical Conditions section of the CRFs;

○ Women of childbearing potential must have been using two medically acceptable methods of contraception, at least one of which must have been a barrier method (e.g., non–hormone-containing intra-uterine device plus condom, spermicidal gel plus condom, diaphragm plus condom), from the time of Screening and for the duration of the study, through at least 1 month following study drug administration. Oral (PO) and topical hormonal contraceptive use, as well as the use of hormone-containing intra-uterine devices, was not permitted due to their increased risk of thromboembolism. Periodic abstinence (e.g., calendar, ovulation, symptom-thermal, post-ovulation methods) and withdrawal were not acceptable methods of contraception;

OR

○ Postmenopausal women must have had no regular menstrual bleeding for at least 1 year before initial dosing and either been over the age of 60 years or had an elevated plasma follicle-stimulating hormone (FSH) level (i.e., > 40 mIU/mL) at Screening; OR

○ Women who reported surgical sterilization (i.e., hysterectomy, tubal ligation, and/or bilateral oophorectomy) must have had the procedure at least 6 months before study drug administration. Surgical sterilization procedures were to be supported with clinical documentation and noted in the Relevant Medical History/Current Medical Conditions of the case report form (CRF).

AND

○ All female subjects must have had a documented negative pregnancy test result at Screening and on Study Day -1. The Screening pregnancy and/or FSH test could have been deferred for subjects that were rescreened;

5) Systolic blood pressure <160 mmHg and diastolic blood pressure < 90 mmHg at Screening and Day -1;

6) The following laboratory values must have been within the normal laboratory reference range within 28 days of Day -1: PT, aPTT, ACT, hemoglobin (Hgb), hematocrit (HCT), and platelet count;

7) The following laboratory values must have been equal to or below 2 times the upper limit of normal range within 28 days of Day -1: aspartate aminotransferase (AST)/alanine aminotransferase (ALT) and total bilirubin;

8) The Screening serum creatinine must have been below 1.5 mg/dL within 28 days of Day -1;

9) Body mass index (BMI) between 19 to 30 kg/m2, inclusive, and body weight of at least 50 kg;

10) Agreed to abstain from alcohol consumption for the duration of the domicile period, and from the use of drugs of abuse for the duration of the study;

11) Able to read and give written informed consent and had signed a consent form approved by the PI’s IRB or Independent Ethics Committee (IEC).

If a patient meets any of the following criteria, he or she is not eligible:

1) Previous use of andexanet or previous participation in the current study;

2) History of abnormal bleeding, signs or symptoms of active bleeding, or risk factors for bleeding;

3) Had a stool specimen that was positive for occult blood within 6 months of study Screening or during the Screening Period;

4) Past or current medical history of thrombosis, and sign or symptom that suggested an increased risk of systemic thrombotic condition or thrombotic event, or recent events that may have increased risk of thrombosis

5) Absolute or relative contraindication to anticoagulation or treatment with apixaban, rivaroxaban, enoxaparin, or edoxaban;

6) Prior consumption (by any route) of one or more doses of aspirin (including baby aspirin), salicylate or subsalicylate, other antiplatelet drugs (e.g., ticlopidine, clopidogrel), non-steroidal anti-inflammatory drugs (NSAIDs), fibrinolytic, or any anticoagulant within 7 days prior to Day -1 or was anticipated to require such drugs during the study;

7) Receipt of (by any route) hormonal contraception, post-menopausal hormone replacement therapy (including over the counter), or testosterone during the 4 weeks prior to Study Day -1 or was anticipated to require such drugs during the study;

8) Family history of or risk factors for a hypercoagulable or thrombotic condition, including one of the following:

○ Factor V Leiden carrier or homozygote;

○ Protein C, S, or AT-III activity below the normal range;

9) History of adult asthma or chronic obstructive pulmonary disease or current regular or as-needed use of inhaled medications;

10) Active hepatitis B virus (HBV), hepatitis c virus (HCV), or human immunodeficiency virus (HIV)-1/2 infection;

11) Use of any drugs that are strong dual inhibitors or inducers of CYP3A4 and Pgp within 7 days prior to Study Day -1 or anticipated need for such drugs during the study;

12) Participation in an investigational drug study within 28 days of Day -1 or Day -1 was within 5 t1/2s of the last dose of the investigational compound;

13) Positive screen for drugs of abuse and/or alcohol at Day -1 that was not explained by a prescription medication that the subject was known to be taking;

14) A medical or surgical condition that could have impaired drug (FXa inhibitor or andexanet) metabolism;

15) Allergy to any of the vehicle ingredients of andexanet: tris, arginine, sucrose, hydrochloric acid, mannitol, and polysorbate 80;

16) Allergy to soy or soy products;

17) Currently breastfeeding or a positive pregnancy test at Screening or Day -1;

18) Any condition that could have interfered with, or for which the treatment might have interfered with, the conduct of the study or interpretation of the study results, or that would have in the opinion of the PI increased the risk of the subject’s participation in the study. This would have included but was not limited to alcoholism, drug dependency or abuse, psychiatric disease, epilepsy, or any unexplained blackouts;

19) Inadequate bilateral venous access per the judgment of the clinical staff and/or the PI;

20) Unwillingness to adhere to the activity requirements of the study.

**Supplementary Text 2: Performed screening test before study enrollment healthy volunteer study:**

Blood was obtained to measure several parameters for the purposes of screening and assessment of eligibility. All test samples were analyzed by the local laboratory. Specifically, these included the following:

• Screening for asymptomatic, pro-thrombotic, hypercoagulable conditions by testing for Factor V Leiden, Protein S, Protein C, and AT-III at the Screening Visit.

• Hepatitis B surface antigen at the Screening Visit.

• Hepatitis C antibody at the Screening Visit.

• HIV-1 and HIV-2 antibodies at the Screening Visit.

• Blood was obtained at Screening, Day -1, Day 8, and Termination Visit (Day 32+3) from all female subjects to determine their pregnancy status (serum pregnancy test and/or FSH as appropriate).

**Supplementary Text 3: Inclusion and exclusion criteria ANNEXA-4 study:**

To be eligible for study enrollment, potential study patients must satisfy all of the following

inclusion criteria:

1) Either the patient or his or her medical proxy has been adequately informed of the nature and

risks of the study and has given written informed consent prior to Screening;

2) The patient must be at least 18 years old at the time of Screening;

3) The patient must have an acute major bleeding episode requiring urgent reversal of

anticoagulation;

4) The patient, for whom the bleeding is intracranial, must have undergone a head CT scan

demonstrating the intracranial bleeding

Note: Patients with bleeding at non-intracranial locations do not require a head CT;

5) Either the patient received a direct or indirect fXa inhibitor within 18 hours prior to the start

of andexanet treatment or believed to be receiving a direct or indirect fXa inhibitor, but the

timing of the last dose is unknown;

6) The patient must agree to comply with the contraception requirements following andexanet

treatment:

Female patients of childbearing potential must use two medically acceptable methods of

contraception unless otherwise dictated by the local ethics committee or Institutional Review

Board. At least one medically acceptable method of contraception must be a barrier method

(e.g., non-hormone containing intra-uterine device plus condom, spermicidal gel plus condom, diaphragm plus condom) from the time of Screening and for at least 2 weeks

following andexanet administration.

For the purpose of this study, all females are considered to be of childbearing potential unless

they are post-menopausal (i.e., at least 1 year since last menses and age >50 years) or

surgically sterile (i.e., tubal ligation, hysterectomy and/or bilateral oophorectomy)

Acute major bleeding is defined by any one of the following:

− Acute bleeding that is potentially life-threatening (e.g., as defined by signs of

hemodynamic compromise such as poor skin perfusion, mental confusion, hypotension,

low urine output);

OR

− Acute bleeding associated with a fall in hemoglobin level by ≥2 g/dL, OR a Hb ≤ 8 g/dL

if no baseline Hb is available OR, in the opinion of the investigator that the patient’s

hemoglobin will fall to ≤ 8 g/dL with resuscitation;

OR

− Acute symptomatic bleeding in a critical area or organ, such as, intraspinal, intraocular,

retroperitoneal, intra-articular or pericardial, or intramuscular with compartment

syndrome.

If a patient meets any of the following criteria, he or she is not eligible:

1) The patient has an expected survival of less than 2 months from causes other than the

bleeding event;

2) The fXa inhibitor being taken by the patient is enoxaparin and the dose is known to be

less than 1 mg/kg/day;

3) The patient is expected to undergo surgery in less than 1 day

Note: Patients requiring minimally invasive procedures (e.g., endoscopy,

bronchoscopy, central lines) that are indicated for diagnostic or therapeutic

reasons eligible to participate in the study;

4) The patient has had acute trauma for which reversal of anticoagulation alone would not

be expected to control or reverse the acute bleeding event;

5) The patient has a known or suspected large blood vessel rupture (e.g., in an advanced

cancer patient or ruptured aneurysm);

6) The patient is known to have taken a VKA (e.g., warfarin) within 7 days prior to

Screening or is receiving dabigatran;

7) The patient is known to have received PCC products (e.g., Kcentra®) or rfVIIa (e.g.,

NovoSeven®) within 28 days of Screening;

8) It is anticipated that the patient will receive unfractionated or low molecular weight

heparin within 24 hours after the start of the andexanet bolus;

9) It is anticipated that the patient will receive PCC, FFP, or rfVIIa within 24 hours after the

start of the andexanet bolus;

10) The patient has a history of a TE, myocardial infarction, DIC, cerebral vascular accident,

transient ischemic attack, unstable angina pectoris, or severe peripheral vascular disease

within 1 month prior to Screening (see Appendix D for DIC scoring algorithm);

11) The patient has severe sepsis or septic shock at the time of Screening (see definition in

Appendix E);

12) If a patient with ICH has any of the following:

− Glasgow coma score < 7

− Intracerebral hematoma volume >30 cc as assessed by the CT volumetric formula

ABC/2

− For subdural hematomas: maximum thickness >10 mm and midline shift >5 mm

− For subarachnoid hematomas, any evidence of hydrocephalus

− Infratentorial ICH location

− Epidural hematomas

− Intraventricular extension of hemorrhage

− Known mRS of >3 prior to ICH;

ABC/2 Formula: A is the greatest hemorrhage diameter by CT, B is the diameter 90°

to A, and C is the approximate number of CT slices with hemorrhage multiplied by the

slice thickness.

13) The patient has been administered whole blood, plasma fractions, or platelets <2 weeks

prior to Screening

Note: Administration of PRBCs is not an exclusion criterion;

14) The patient has a known history of anti-phospholipid antibody syndrome or lupus

anticoagulant antibodies;

15) The patient has a known history of an inhibitor to coagulation factors II, VII, IX, or X

(other than treatment with an anti-fXa inhibitor drug);

16) The patient is pregnant or a lactating female;

17) The patient was treated with an investigational drug <30 days prior to Screening

***Supplementary Table 1. Rating system for hemostatic efficacy in ICH***

| **Bleeding location** | **Excellent (effective) hemostatic efficacy** | **Good (effective) hemostatic efficacy** | **Poor/none (not effective) hemostatic efficacy** |
| --- | --- | --- | --- |
| **Visible** | Cessation of bleeding ≤1 hour after the end of infusion and no plasma, coagulation factor or blood products | Cessation of bleeding between >1 and ≤4 hours after end of infusion and ≤2units plasma, coagulation factor or blood products | Cessation of bleeding >4hours after end of the infusion of and/or >2 units plasma, coagulation factor or blood products |
| **Muscular/skeletal** | Pain relief or no increase in swelling or unequivocal improvement in objective signs of bleeding ≤1hour after the end of infusion; and the condition has not deteriorated during 12-h period | Pain relief or no increase in swelling or unequivocal improvement in objective signs of bleeding >1 and ≤4hours after end of infusion; and the condition has not deteriorated during the 12-h period | No improvement by 4hours after end of infusion and/or condition has deteriorated during the 12h period |
| **Intracerebral hematoma** | ≤20% increase in hematoma volume compared to baseline on a repeat CT or MRI scan  performed at both the 1 and 12 hour post infusion andexanet | >20% but ≤35% increase in hematoma volume compared to baseline on a repeat CT or MRI scan at +12-hour time point | >35% increase in hematoma volume on a CT or MRI compared to baseline on a repeat CT or MRI scan at +12-hour time point |
| **Subarachnoid bleed** | ≤20% increase in maximum thickness using the  most dense area on the follow-up vs baseline at both the 1 and 12 hour post infusion time points | >20% but <35% increase in maximum thickness using the most dense area on the follow-up at +12h vs baseline | >35% increase in maximum thickness using the most dense area on the +12h vs at baseline |
| **Subdural hematoma** | ≤20% increase in maximum thickness at both  the 1 and 12 hour post infusion assessments compared to baseline | >20% but < 35% increase in maximum thickness at +12h compared to baseline | >35% increase in maximum thickness at  +12h compared to baseline |
| **Pericardial** | No increase in size of pericardial effusion on repeat echocardiogram done within 12hours of the end of infusion | <10% increase in the size of pericardial effusion on repeat echocardiogram done within 12hours of the end of infusion | 10% or more increase in size of pericardial effusion on repeat echocardiogram done within 12hours of the end of infusion |
| **Intra-spinal** | No increase in hematoma size on repeat CT or MRI scan done within 12hours of the end of infusion | <10% increase in hematoma size on repeat CT or MRI scan done within 12hours of the end of infusion | 10% or more increase in hematoma size on repeat CT or MRI scan done within 12hours of the end of infusion |
| **GI, Urinary or non-visible bleeding not described above** | ≤10% decrease in both corrected hemoglobin/hematocrit at 12hours compared to baseline | >10% to ≤20% decrease in both corrected hemoglobin/hematocrit at 12 hours compared to baseline | >20% decrease in both corrected hemoglobin/hematocrit |

***Supplementary Table 2. Enoxaparin Standard Preparation***

| **Solution** | **Enoxaparin Concentration (IU/mL)** | | | | | | |
| --- | --- | --- | --- | --- | --- | --- | --- |
|  | **1.00** | **0.75** | **0.50** | **0.25** | **0.10** | **0.05** | **0.00** |
| **2.0 IU/mL Stock (µL)** | 200 | 150 | 100 | 50 | 20 | 10 | 0 |
| **Pooled Normal Plasma (µL)** | 200 | 250 | 300 | 350 | 380 | 390 | 400 |

***Supplementary Table 3. Preparation of Enoxaparin Controls***

| **Control type** | **Enoxaparin (IU/mL)** | **PNP Volume (µL)** | **2.0 IU/mL Stock Volume (µL)** |
| --- | --- | --- | --- |
| **CTL3** | 0.80 | 960 | 640 |
| **CTL2** | 0.50 | 1200 | 400 |
| **CTL1** | 0.25 | 1400 | 200 |
